# Crystal structure, Hirshfeld surface, DFT and mol­ecular docking studies of 2-{4-[(*E*)-(4-acetylphen­yl)diazen­yl]phen­yl}-1-(5-bromo­thio­phen-2-yl)ethanone; a compound with bromine⋯oxygen-type contacts

**DOI:** 10.1107/S2056989024010776

**Published:** 2024-11-22

**Authors:** S. Santhosh Kumar, H.T Srinivasa, M. Harish Kumar, H. C. Devarajegowda, B. S. Palakshamurthy

**Affiliations:** a Department of PG Studies and Research in Physics Albert Einstein Block UCS Tumkur University, Tumkur Karnataka-572103 India; b Raman Research Institute, C V Raman Avenue Sadashivanagar Bangalore Karnataka India; c Department of Physics Yuvaraja's College University of Mysore,Mysore 570005 Karnataka India; Universidade Federal do ABC, Brazil

**Keywords:** crystal structure, azo­benzene, mol­ecular docking, C—Br⋯O=C contact, DFT and Hirshfeld surface

## Abstract

The title compound, C_19_H_13_BrN_2_O_3_S, a non-liquid crystal mol­ecule, crystallizes in the ortho­rhom­bic system, space group *Pna*2_1_. The torsion angles associated with ester and azo groups are −177.0 (4)°, -anti-periplanar, and 179.0 (4)°, +anti-periplanar, respectively. The packing is consolidated by a weak C—Br⋯O=C contact, forming infinite chains running along the [001] direction. A Hirshfeld surface analysis revealed that the major contributions to the crystal surface are from H⋯H, C⋯H/H⋯C, O⋯H/H⋯O, Br⋯H/H⋯Br and S⋯H/H⋯S inter­actions. The computed three-dimensional energy inter­actions using the basis set B3LYP\631-G(d,p) show that *E_dis_* (217.6 kJ mol^−1^) is the major component in the structure. The DFT calculations performed at the B3LYP/6–311+ G(d,p) level indicate that the energy gap between HOMO and LUMO is 3.6725 (2) eV. The mol­ecular electrostatic potential (MEP) map generated supports the existence of the Br⋯O type contact, formed between the electrophilic site of the bromine atom and the nucleophilic site of the ketonic oxygen atom. The mol­ecular docking between the ligand and the *Mycobacterium Tuberculosis* (PDB ID:1HZP) receptor shows a good binding affinity value of −8.5 kcal mol^−1^.

## Abstract 2

The crystal structure of the title non-liquid crystal compound is consolidated by C—Br⋯O=C type contacts running continuously along the [001] direction.

## 1. Chemical context

Azo­benzenes are a class of mol­ecules having high structural similarity with stilbenes, which exhibit anti­bacterial and anti­fungal activity (Piotto *et al.*, 2013 ▸). They are capable of regulating the structure and function of various biological mol­ecules, including proteins, nucleic acids, lipids and peptides (Mulatihan *et al.*, 2020 ▸). Azo­benzene derivatives exhibit biological activities such as anti­oxidant, anti­viral and anti­microbial properties (Ventura & Wiedman, 2021 ▸; Kaur & Narasimhan, 2018 ▸; Peddie & Abell, 2019 ▸). Azo­benzene-based polymeric nanocarriers are biocompatible materials and they can accelerate drug-release systems in biological tissues (Londoño-Berrío *et al.*, 2022 ▸). Inter­estingly thio­phene-based derivatives exhibit significant anti-leishmanial activity (Félix *et al.*, 2016 ▸) and anti­malarial activity (Akolkar *et al.*, 2022 ▸). The thio­phene derivatives with anti­tubulin properties are potential materials for the treatment of cancer and Alzheimer’s and Parkinson’s diseases (Romagnoli *et al.*, 2007 ▸). The thio­phene-thia­zole derivatives are a class of materials having anti­tubulin properties and they can also be used as anti-breast cancer agents (Al-Said *et al.*, 2011 ▸). Azo­benzenes, and their derivatives in a *trans* confirmation, are medically important. They are also useful in industry because of their inter­esting photoswitch properties and have therefore been widely explored as photoresponsive compounds in materials science (Li *et al.*, 2024 ▸). Keeping photoswitching properties in mind, we have planed to use the tris­(azo­benzene) as a core group in the construction of liquid-crystal materials. Hence, we developed the title mol­ecule, (I), to analyse the mol­ecular properties both experimentally and theoretically and present the results herein.
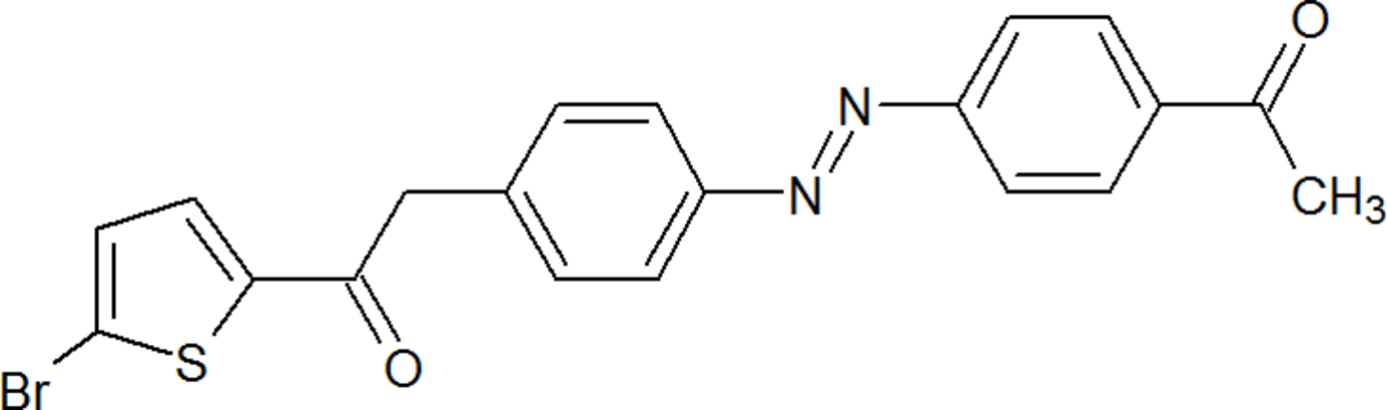


## 2. Structural commentary

**Figure 1:**
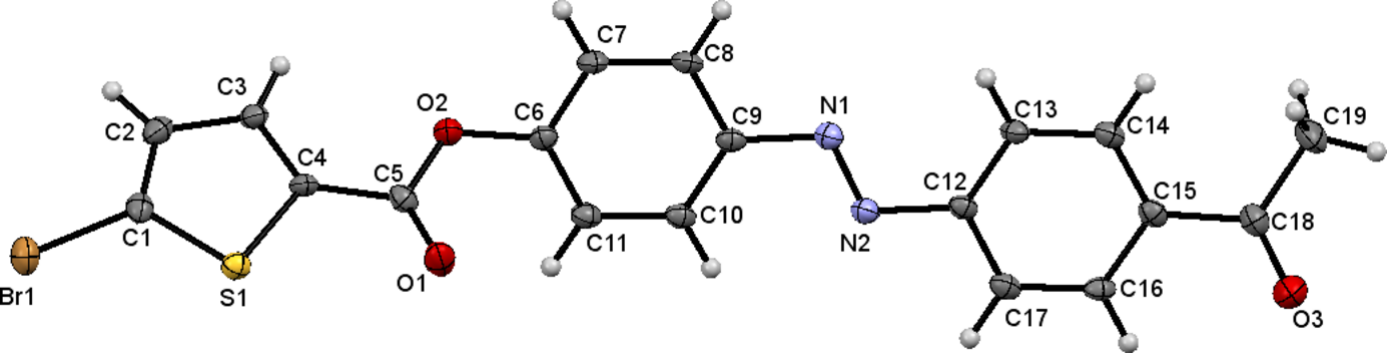
Mol­ecular structure of the title compound, showing displacement ellipsoids drawn at the 50% probability level.

The mol­ecular structure of compound (I) is shown in Fig. 1 ▸. In the mol­ecule, the dihedral angles between the five-membered thio­phene and the benzene and phenyl­ethanone rings are 1.6 (2) and 54.0 (2)°, respectively, while that between the aromatic rings is 52.5 (2)°. The torsion angles associated with the ester (C—C—O—C) and azo (C—N=N—C) groups in the mol­ecule are -anti-periplanar [−177.0 (4)°] and +anti-periplanar [179.0 (4)°], respectively. Intra­molecular C13—H13⋯N1, C10—H10⋯N2, and C16—H16⋯O3 inter­actions (Table 1 ▸) stabilize the mol­ecular structure.

## 3. Supra­molecular features

**Figure 2:**
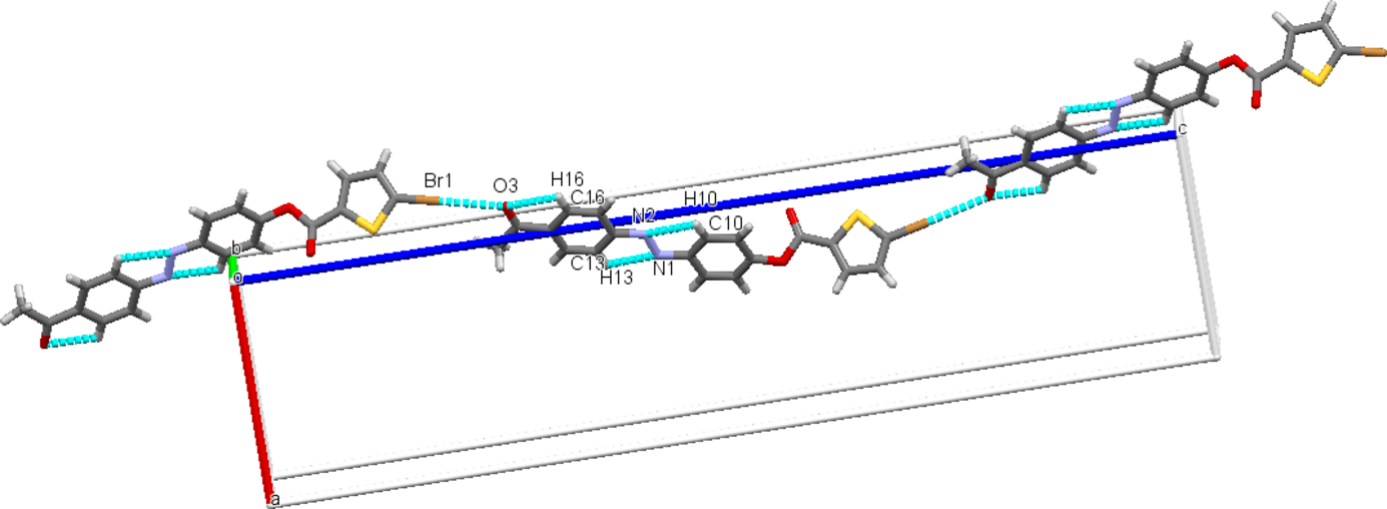
Mol­ecular packing of the title compound, Dashed lines indicate C—Br⋯O=C intra­molecular contacts.

The crystal packing is consolidated by C1—Br1⋯O3=C18 type inter­action with a Br1⋯O3 distance 3.014 (2) Å, forming infinite chains running in the [001] direction as shown in Fig. 2 ▸.

## 4. Database survey

A search of the Cambridge Structural Database (CSD, version 5.42, update of November 2020; Groom *et al.*, 2016 ▸) for mol­ecules containing the *trans*­(azo­benzene) fragment resulted in two matches with CSD refcodes APOPUR, APOQAY and APOQEC (Soegiarto *et al.*, 2010 ▸). In APOPUR, 4,4′-azo­benzene­disulfonate is associated with two azo­benzene mol­ecules, one of which is disordered, with the another having a tris conformation. The torsion angles at the azo and azo­benzene groups are180.00 and −179.96°. In APOQAY, the 4,4′- di­benzyl­disulfonate moiety is associated with two azo­benzene fragments in which the torsion angles at the azo groups are 180.0 and178.39° and have a tris conformation. In APOQEC, the 4,4′-stilbenedi­sulfonate is associated with two azo­benzene moieties with torsion angles at the azo group of 180 and −177.93 (2)°. These are comparable with the torsion angle at the azo group of 179.0 (4)° in the tris­(azo­benzene) fragment of the title mol­ecule. In all these compounds, the torsion angles at the azo group are *anti.*.

## 5. Hirshfeld surface analysis and inter­action energies

**Figure 3:**
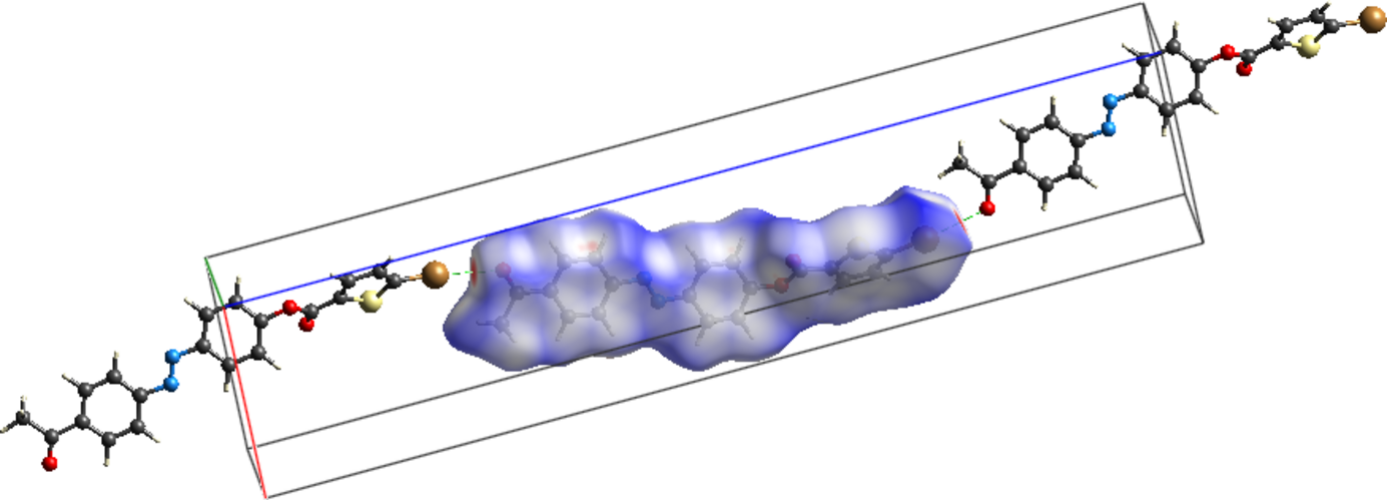
Hirshfeld surface mapped over *d*_norm_ with red spots corresponding to short C—Br⋯O=C contacts.

**Figure 4:**
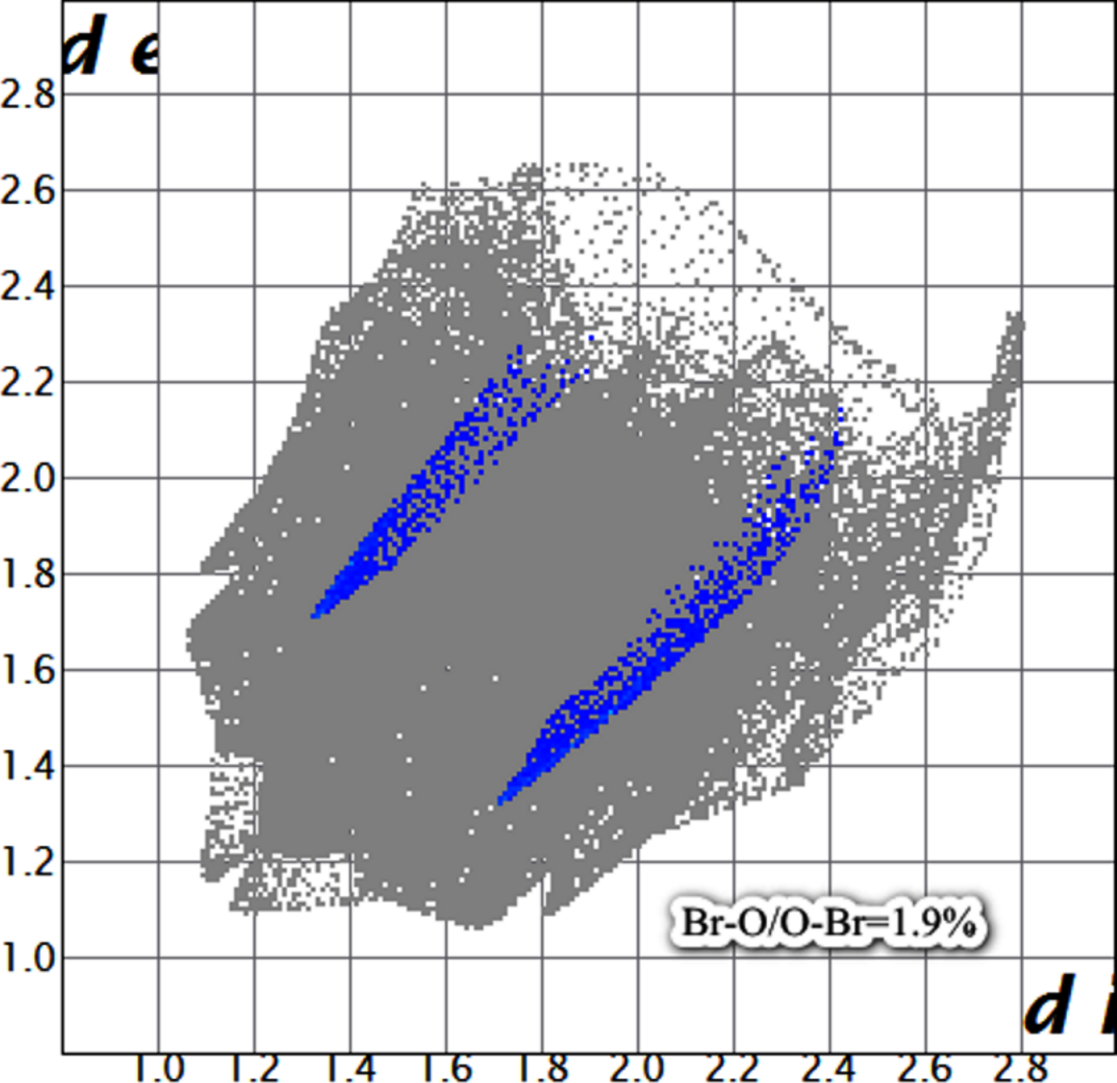
The two-dimensional fingerprint plots (spikes) showing the halogen–oxygen inter­actions.

**Figure 5:**
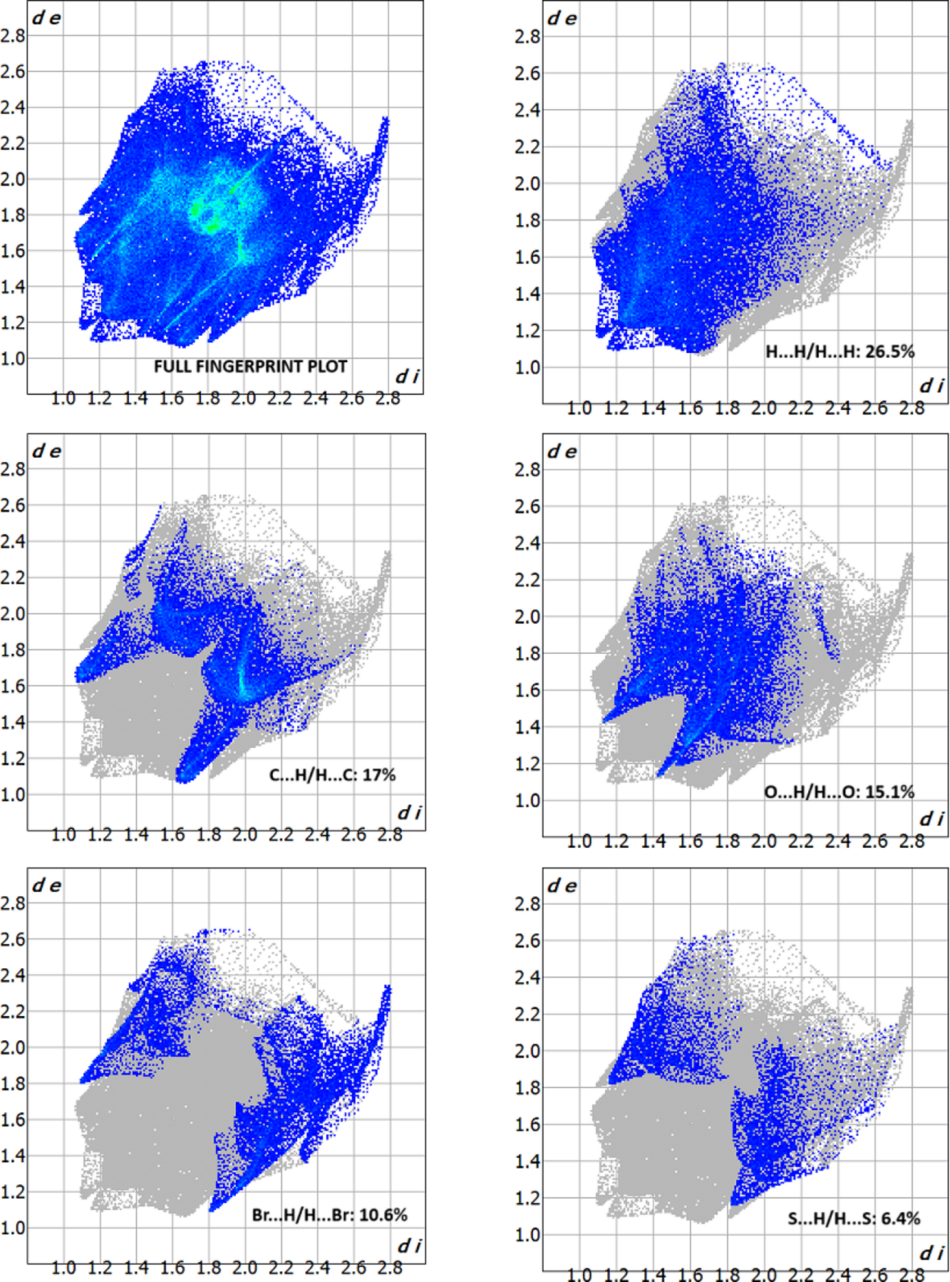
The two-dimensional fingerprint plots showing all inter­actions and those delineated into H⋯H, C⋯H/H⋯C, O⋯H/ H⋯O, Br⋯H/H⋯Br and S⋯H/H⋯S inter­actions.

A Hirshfeld surface analysis was carried out to qu­antify the various inter­molecular inter­actions contributed to the crystal using *CrystalExplorer17.5* (Turner *et al.*, 2017 ▸). Fig. 3 ▸ illustrates the Hirshfeld surface mapped over *d*_norm_ with red spots corresponding to short C—Br⋯O=C contacts. It is an additional confirmation of the presence of Br⋯O type contacts (*i.e* halogen–oxygen type). In Fig. 4 ▸, the sharp spikes in the Br⋯O/O⋯Br fingerprint plot indicate the existence of a weak halogen⋯oxygen inter­action in the mol­ecular structure. Although it is a weak inter­action, the packing of the title compound is consolidated by Br⋯O inter­actions and no other inter­actions are present. The fingerprint plots showing all inter­actions and those delineated into H⋯H, C⋯H/H⋯C, O⋯H/ H⋯O, Br⋯H/H⋯Br and S⋯H/H⋯S inter­actions as shown in Fig. 5 ▸.

**Figure 6:**
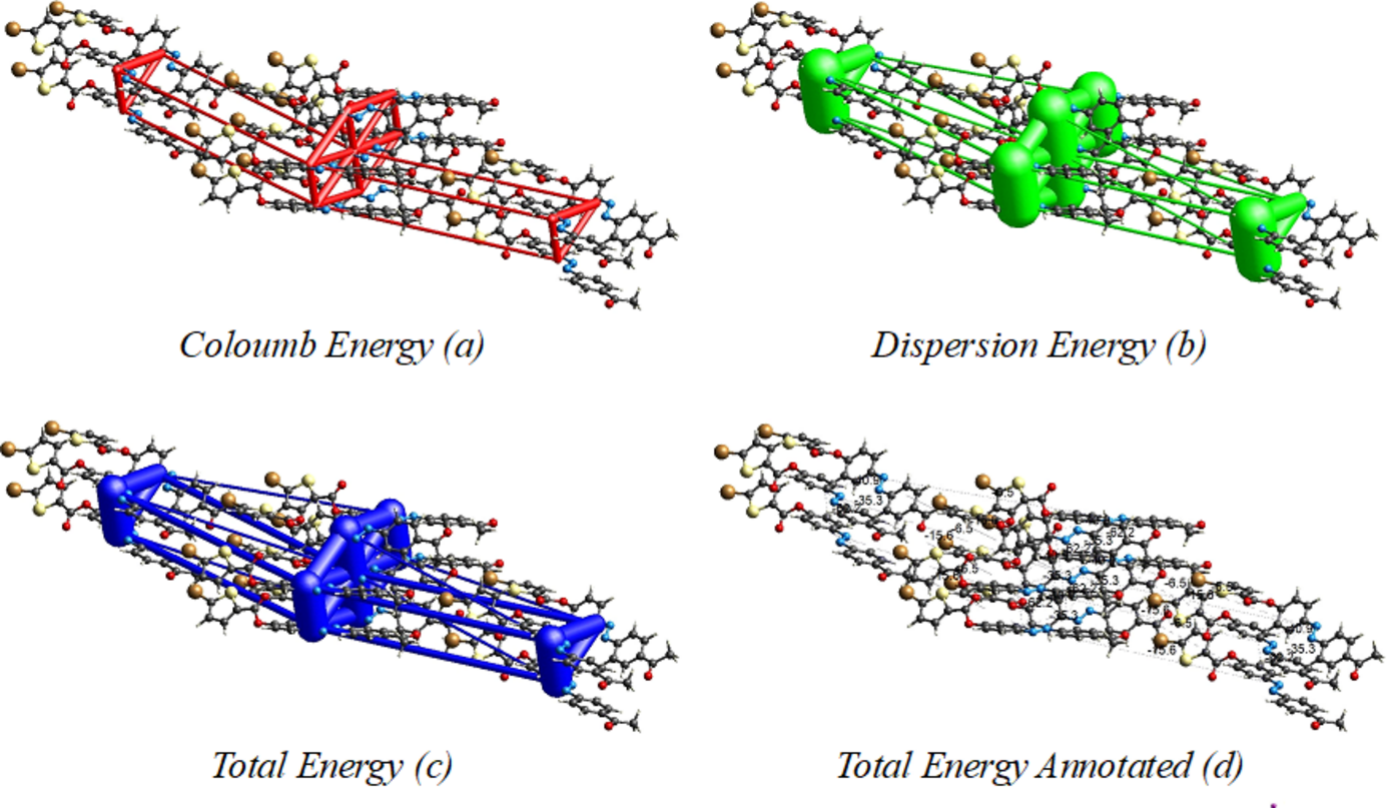
The energy frameworks for the inter­action energies in the title compound (*a*) Coulombic energy, (*b*) dispersion energy, (*c*) total energy and (*d*) total energy annotated.

Three-dimensional energy inter­actions were computed for the title compound using the B3LYP\631-G(d,p) basis set, indicating that the *E*_dis_ = 217.6 kJ mol^−1^ is the major component, the others being *E*_ele_ = 55.3 kJ mol^−1^, *E*_pol_ =11.6 kJ mol^−1^, *E*_rep_ = 148.6 kJ mol^−1^ with a total inter­action energy *E_tot_* of 164.7 kJ mol^−1^. The energy frameworks for the inter­action energies are shown in Fig. 6 ▸.

## 6. DFT Studies

**Figure 7:**
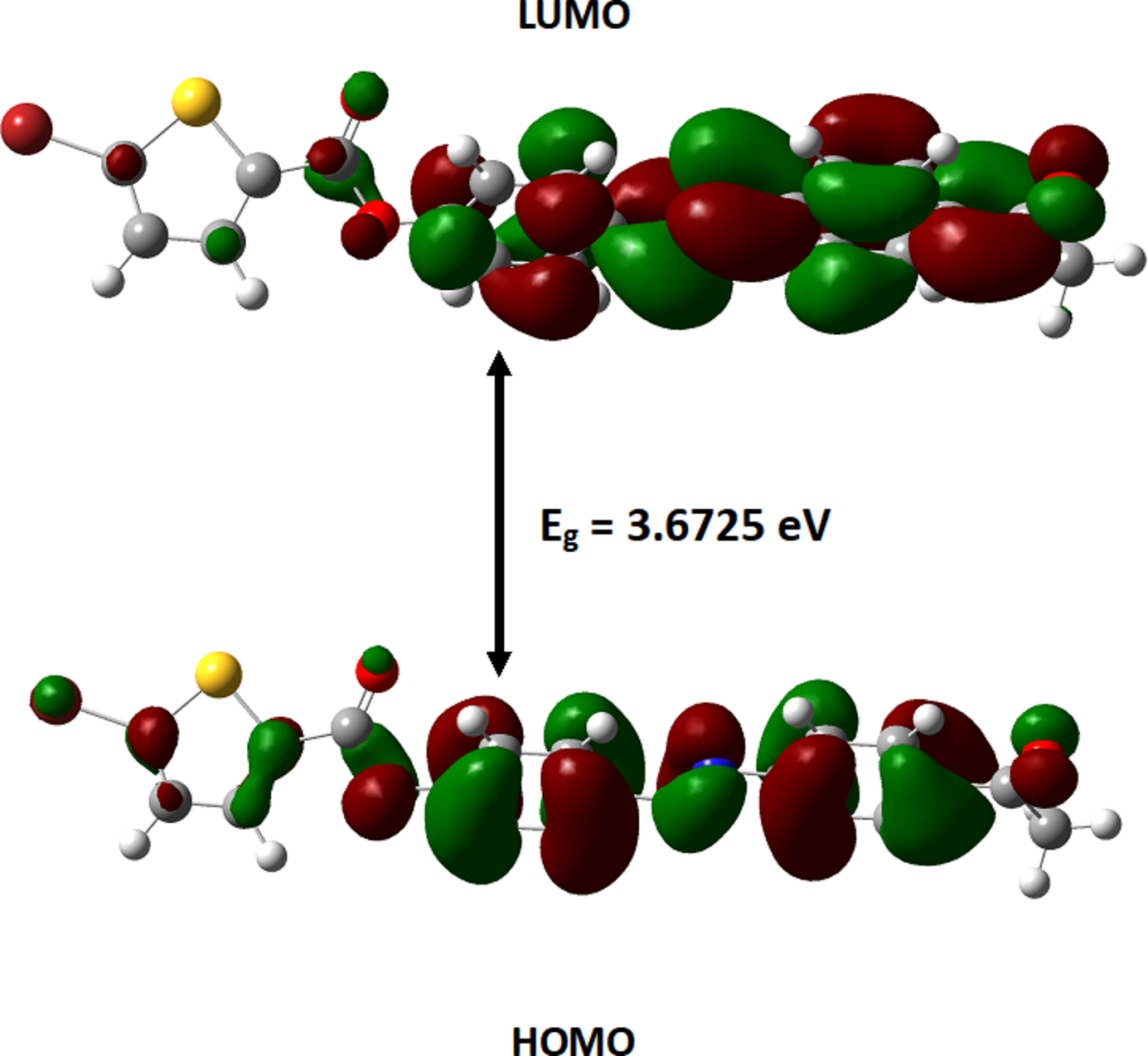
The HOMO and LUMO orbitals of the title mol­ecule.

The mol­ecular structure of the title compound in the gas phase was optimized using density functional theory with the standard B3LYP method with the basis set 6-311++G(d,p). The input files were prepared from the CIF file using *Mercury* (Macrae *et al.*, 2020 ▸) and *Gauss View* 6.0 (Frisch *et al.*, 2009 ▸). The electron density distribution in the frontier mol­ecular orbital are shown in Fig. 7 ▸. The highest occupied mol­ecular orbital (HOMO) is −6.7179 eV) and the lowest unoccupied mol­ecular orbital (LUMO) is −3.0454 eV) with an energy gap of 3.6725 eV. The electrophilicity index (ω) is 6.489 eV, which indicates the mol­ecule is highly reactive.

**Figure 8:**
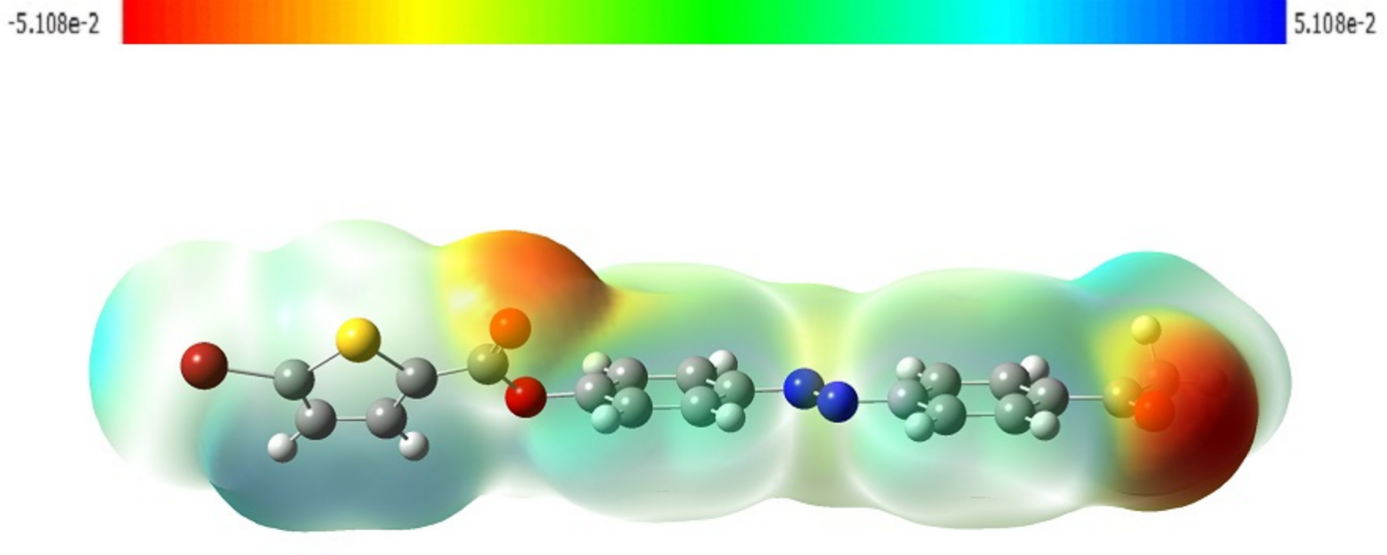
The mol­ecular electrostatic potential (MEP) surface of the title mol­ecule.

The mol­ecular electrostatic potential (MEP) map predicts the reactive sites for electrophilic and nucleophilic attack present in the mol­ecule. In the crystal, the mol­ecular charge distribution is between −5.108 × 10^−2^ and +5.108 × 10^−2^ and is governed by the MEP (Fig. 8 ▸). The red colour around the oxygen atoms of the ester group and ketone group in the mol­ecule indicates nucleophilic sites and the pale-blue colour around the bromine atom indicate the active electrophilic site. In the crystal, the Br⋯O type contact formed between the electrophilic site of the bromine atom and the nucleophilic site of the ketonic oxygen atom connects the mol­ecule into infinite chains along the *c*-axis direction.

## 7. Mol­ecular docking

**Figure 9:**
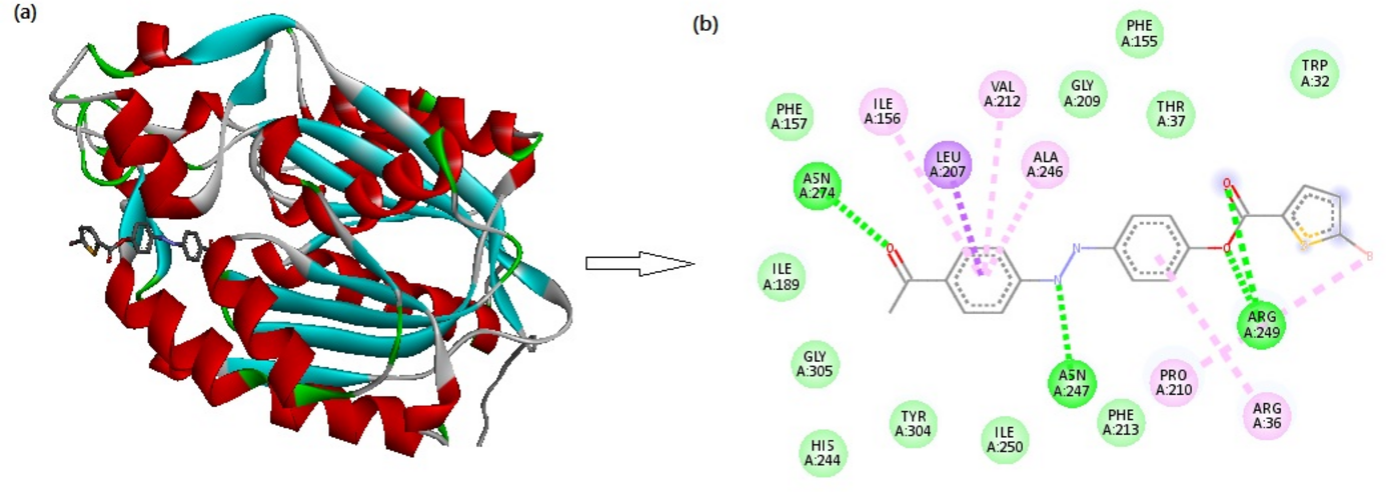
Three-dimensional and two-dimensional views of various inter­actions between the title compound (ligand) and *Myobacterium Tuberculosis* bacteria (PDB ID:1HZP; receptor protein).

The mol­ecular docking studies using *AutoDock* tools (Huey *et al.*, 2012 ▸) were carried out to calculate the degree of binding affinity between the synthesised ligand and the receptor protein of *Mycobacterium Tuberculosis* bacteria (PDB ID:1HZP). It is found that, among the several inter­actions with the target protein, four conventional hydrogen-bonding inter­actions are seen between four moieties, which are formed by the oxygen and nitro­gen atoms of the ketonic, ester and azo groups, as shown in Fig. 9 ▸. The centroids of the ­benzene rings (*Cg*1, C6–C11 and *Cg*2, C12–C17) attack different amino acid groups (ILE A:156, VAL A:212, ALA A:246 and ARG A:36). *PLATON* (Spek, 2020 ▸) indicates very weak π–π stacking between these rings with *Cg*1⋯*Cg*2 separations of 5.609 (3) to 5.616 (2) Å; these stacking inter­actions were able to attack the aforementioned amino acids of the protein. In addition to these, the bromine atom in the mol­ecule has an affinity with another amino acid group (PRO A:210) through nucleophilic attacks. In total, the ligand shows a good binding affinity value of −8.5 kcal mol^−1^.

## 8. Synthesis and crystallization

5-Bromo­thio­phene-2-carb­oxy­lic acid (1 eq, 0.207 g), (*E*)-1-{4-[(4-hy­droxy­phen­yl)diazen­yl]phen­yl}ethan-1-one (1 eq, 0.240 g), di­cyclo­hexyl­carbodi­imide (1.2 eq) and a catalytic amount of di­methyl­amino­pyrimidine were stirred in dry di­chloro­methane at room temperature overnight. Completion of the reaction was verified by thin layer chromatography on silica gel on an aluminium plate with di­chloro­methane as the mobile phase. After completion of the reaction, the whole reaction mass was subjected to column chromatography with silica gel and a 1:9 ratio of petroleum ether and di­chloro­methane as eluent. The solvent was evaporated under vacuum, and the crude product was recrystallized from pure chloro­form to obtain single crystals suitable for single-crystal X-ray studies. The compound is orange in colour, m.p. 469 K, mol­ecular weight is 429.29. Elemental analysis, calculated: C, 53.16; H, 3.05; Br, 18.61; N, 6.53; O, 11.18; S, 7.47; found: C, 53.19; H, 3.09; N, 6.60; S, 7.52%. ^1^H NMR: (500 MHz, CDCl_3_) δ/ppm, 8.13–7.97 (*m*, 6H, Ar-H), 7.76 (*d*, 1H, *J* = 6 Hz, Ar-H), 7.43 (*m*, 2H, Ar-H), 7.18 (*m*, 1H, Ar-H), 1.57 (*s*, 3H, COCH_3_) ppm. ^13^C NMR (CDCl_3_) δ/ppm: 197.5, 159.1, 154.9, 152.8, 138.5, 135.3, 131.4, 122.9, 26.9.

## 9. Refinement

Crystal data, data collection and structure refinement details are summarized in Table 2 ▸. All the H-atoms were positioned with idealized geometry and refined using a riding model with C—H = 0.95–0.98 Å and *U*_iso_(H) = 1.2*U*_eq_(C) or 1.5*U*_eq_(methyl C). The crystal studied was refined as an inversion twin.

## Supplementary Material

Crystal structure: contains datablock(s) I. DOI: 10.1107/S2056989024010776/ee2010sup1.cif

Structure factors: contains datablock(s) I. DOI: 10.1107/S2056989024010776/ee2010Isup3.hkl

Supporting information file. DOI: 10.1107/S2056989024010776/ee2010Isup3.cml

CCDC reference: 2401081

Additional supporting information:  crystallographic information; 3D view; checkCIF report

## Figures and Tables

**Table 1:** Hydrogen-bond geometry (Å, °)

*D*—H⋯*A*	*D*—H	H⋯*A*	*D*⋯*A*	*D*—H⋯*A*
C13—H13⋯N1	0.95	2.47	2.722 (6)	95
C16—H16⋯O3	0.95	2.49	2.788 (6)	98
C10—H10⋯N2	0.95	2.45	2.705 (6)	95

**Table 2:** Experimental details

Crystal data	
Chemical formula	C_19_H_13_BrN_2_O_3_S
*M*_r_	429.28
Crystal system, space group	Orthorhombic, *Pna*2_1_
Temperature (K)	120
*a*, *b*, *c* (Å)	10.5425 (13), 3.8532 (5), 42.419 (4)
*V* (Å^3^)	1723.1 (4)
*Z*	4
Radiation type	Mo *K*α
μ (mm^−1^)	2.53
Crystal size (mm)	0.41 × 0.28 × 0.18

Data collection	
Diffractometer	Bruker *SMART* APEXII CCD
Absorption correction	Multi-scan (*SADABS*; Krause *et al.*, 2015 ▸)
*T*_min_, *T*_max_	0.431, 0.633
No. of measured, independent and observed [*I* > 2σ(*I*)] reflections	36482, 5305, 5216
*R*_int_	0.029
(sin θ/λ)_max_ (Å^−1^)	0.716

Refinement	
*R*[*F*^2^ > 2σ(*F*^2^)], *wR*(*F*^2^), *S*	0.037, 0.091, 1.23
No. of reflections	5305
No. of parameters	237
No. of restraints	1
H-atom treatment	H-atom parameters constrained
Δρ_max_, Δρ_min_ (e Å^−3^)	1.12, −1.54
Absolute structure	Refined as an inversion twin
Absolute structure parameter	0.021 (12)

Computer programs: *APEX2* and *SAINT* (Bruker, 2014 ▸), *SHELXS97* (Sheldrick, 2008 ▸), *SHELXL2018/3* (Sheldrick, 2015 ▸) and *Mercury* (Macrae *et al.*, 2020 ▸).

## supplementary crystallographic information

### 2-{4-[(E)-(4-Acetylphenyl)diazenyl]phenyl}-1-(5-bromothiophen-2-yl)ethanone Crystal data

**Table:**

C_19_H_13_BrN_2_O_3_S	*D*_x_ = 1.655 Mg m^−^^3^
*M**_r_* = 429.28	Melting point: 469 K
Orthorhombic, *P**n**a*2_1_	Mo *K*α radiation, λ = 0.71074 Å
Hall symbol: P 2c -2n	Cell parameters from 5305 reflections
*a* = 10.5425 (13) Å	θ = 2.8–30.1°
*b* = 3.8532 (5) Å	µ = 2.53 mm^−^^1^
*c* = 42.419 (4) Å	*T* = 120 K
*V* = 1723.1 (4) Å^3^	Prism, orange
*Z* = 4	0.41 × 0.28 × 0.18 mm
*F*(000) = 864	

### 2-{4-[(E)-(4-Acetylphenyl)diazenyl]phenyl}-1-(5-bromothiophen-2-yl)ethanone Data collection

**Table:**

Bruker SMART APEXII CCD diffractometer	5305 independent reflections
Radiation source: fine-focus sealed tube	5216 reflections with *I* > 2σ(*I*)
Graphite monochromator	*R*_int_ = 0.029
Detector resolution: 0.99 pixels mm^-1^	θ_max_ = 30.6°, θ_min_ = 2.9°
φ and Ω scans	*h* = −15→15
Absorption correction: multi-scan (SADABS; Krause *et al.*, 2015)	*k* = −5→5
*T*_min_ = 0.431, *T*_max_ = 0.633	*l* = −60→60
36482 measured reflections	

### 2-{4-[(E)-(4-Acetylphenyl)diazenyl]phenyl}-1-(5-bromothiophen-2-yl)ethanone Refinement

**Table:**

Refinement on *F*^2^	Secondary atom site location: difference Fourier map
Least-squares matrix: full	Hydrogen site location: inferred from neighbouring sites
*R*[*F*^2^ > 2σ(*F*^2^)] = 0.037	H-atom parameters constrained
*wR*(*F*^2^) = 0.091	*w* = 1/[σ^2^(*F*_o_^2^) + (0.0229*P*)^2^ + 3.5453*P*] where *P* = (*F*_o_^2^ + 2*F*_c_^2^)/3
*S* = 1.23	(Δ/σ)_max_ < 0.001
5305 reflections	Δρ_max_ = 1.12 e Å^−^^3^
237 parameters	Δρ_min_ = −1.54 e Å^−^^3^
1 restraint	Absolute structure: Refined as an inversion twin
0.132 constraints	Absolute structure parameter: 0.021 (12)
Primary atom site location: structure-invariant direct methods	

### 2-{4-[(E)-(4-Acetylphenyl)diazenyl]phenyl}-1-(5-bromothiophen-2-yl)ethanone Special details

**Table:**

Geometry. All esds (except the esd in the dihedral angle between two l.s. planes) are estimated using the full covariance matrix. The cell esds are taken into account individually in the estimation of esds in distances, angles and torsion angles; correlations between esds in cell parameters are only used when they are defined by crystal symmetry. An approximate (isotropic) treatment of cell esds is used for estimating esds involving l.s. planes.
Refinement. Refined as a 2-component inversion twin

### 2-{4-[(E)-(4-Acetylphenyl)diazenyl]phenyl}-1-(5-bromothiophen-2-yl)ethanone Fractional atomic coordinates and isotropic or equivalent isotropic displacement parameters (Å^2^)

**Table:**

	*x*	*y*	*z*	*U*_iso_*/*U*_eq_	
Br1	0.72359 (4)	0.43138 (12)	0.72407 (2)	0.02567 (11)	
S1	0.65138 (10)	0.3319 (3)	0.65409 (3)	0.01799 (19)	
O2	0.7849 (3)	0.5368 (9)	0.56998 (7)	0.0209 (6)	
O1	0.6053 (3)	0.2541 (11)	0.58516 (8)	0.0261 (7)	
O3	0.3469 (4)	0.5729 (11)	0.29358 (10)	0.0296 (9)	
C2	0.8653 (4)	0.6201 (12)	0.66779 (11)	0.0213 (9)	
H2	0.934208	0.714315	0.679421	0.026*	
N1	0.6770 (4)	0.4543 (10)	0.44152 (9)	0.0180 (7)	
N2	0.5674 (3)	0.5376 (10)	0.43322 (9)	0.0181 (7)	
C3	0.8591 (4)	0.6110 (12)	0.63421 (10)	0.0173 (8)	
H3	0.924322	0.694646	0.620752	0.021*	
C6	0.7507 (4)	0.5120 (11)	0.53813 (10)	0.0167 (7)	
C18	0.4512 (4)	0.4762 (13)	0.30191 (11)	0.0213 (8)	
C8	0.8114 (4)	0.3473 (12)	0.48602 (10)	0.0177 (8)	
H8	0.870799	0.246549	0.471880	0.021*	
C17	0.4310 (4)	0.6460 (12)	0.38941 (10)	0.0186 (8)	
H17	0.372427	0.744592	0.403888	0.022*	
C11	0.6381 (4)	0.6597 (12)	0.52722 (10)	0.0184 (8)	
H11	0.580744	0.768563	0.541366	0.022*	
C13	0.6316 (4)	0.3619 (12)	0.37891 (10)	0.0177 (8)	
H13	0.709212	0.267351	0.386355	0.021*	
C14	0.6021 (4)	0.3488 (12)	0.34710 (10)	0.0178 (8)	
H14	0.659950	0.245795	0.332706	0.021*	
C16	0.4025 (4)	0.6309 (11)	0.35762 (11)	0.0169 (8)	
H16	0.323815	0.720403	0.350343	0.020*	
C9	0.6980 (4)	0.4834 (11)	0.47477 (10)	0.0161 (7)	
C7	0.8372 (4)	0.3598 (12)	0.51804 (10)	0.0181 (8)	
H7	0.913759	0.264461	0.526070	0.022*	
C1	0.7600 (4)	0.4768 (12)	0.68108 (10)	0.0199 (8)	
C4	0.7478 (4)	0.4672 (12)	0.62360 (10)	0.0158 (7)	
C12	0.5465 (4)	0.5149 (11)	0.39993 (10)	0.0159 (7)	
C10	0.6122 (4)	0.6435 (11)	0.49528 (10)	0.0169 (7)	
H10	0.535967	0.741241	0.487273	0.020*	
C5	0.7016 (4)	0.4050 (11)	0.59146 (10)	0.0182 (8)	
C15	0.4873 (4)	0.4867 (11)	0.33604 (10)	0.0175 (8)	
C19	0.5478 (5)	0.3457 (17)	0.27848 (12)	0.0320 (11)	
H19A	0.578978	0.117756	0.285185	0.048*	
H19B	0.618949	0.509129	0.277391	0.048*	
H19C	0.508350	0.325653	0.257643	0.048*	

### 2-{4-[(E)-(4-Acetylphenyl)diazenyl]phenyl}-1-(5-bromothiophen-2-yl)ethanone Atomic displacement parameters (Å^2^)

**Table:**

	*U*^11^	*U*^22^	*U*^33^	*U*^12^	*U*^13^	*U*^23^
Br1	0.0321 (2)	0.02593 (19)	0.01893 (16)	−0.00111 (18)	−0.0019 (2)	0.0010 (2)
S1	0.0169 (4)	0.0172 (4)	0.0199 (4)	−0.0016 (4)	0.0006 (4)	−0.0011 (4)
O2	0.0166 (14)	0.0271 (16)	0.0189 (14)	−0.0018 (13)	0.0006 (11)	−0.0014 (12)
O1	0.0262 (17)	0.0306 (19)	0.0214 (15)	−0.0109 (15)	0.0015 (13)	−0.0022 (15)
O3	0.0244 (17)	0.038 (2)	0.0266 (18)	0.0038 (16)	−0.0022 (15)	−0.0007 (16)
C2	0.0172 (19)	0.022 (2)	0.025 (2)	0.0016 (16)	−0.0047 (15)	−0.0030 (16)
N1	0.0166 (15)	0.0185 (16)	0.0189 (16)	0.0009 (13)	0.0018 (13)	0.0005 (14)
N2	0.0151 (15)	0.0209 (17)	0.0183 (16)	−0.0007 (14)	0.0023 (13)	−0.0016 (13)
C3	0.0128 (17)	0.020 (2)	0.0193 (18)	−0.0001 (15)	−0.0004 (14)	0.0010 (15)
C6	0.0141 (17)	0.0180 (18)	0.0179 (17)	−0.0029 (14)	0.0008 (14)	−0.0004 (15)
C18	0.024 (2)	0.022 (2)	0.0188 (19)	0.0004 (17)	0.0018 (16)	−0.0020 (16)
C8	0.0114 (16)	0.0192 (19)	0.0225 (19)	−0.0002 (15)	0.0046 (14)	−0.0009 (15)
C17	0.0166 (18)	0.0174 (18)	0.0217 (19)	−0.0016 (16)	0.0043 (15)	−0.0015 (15)
C11	0.0156 (18)	0.0177 (18)	0.0218 (18)	0.0006 (15)	0.0028 (14)	−0.0037 (15)
C13	0.0134 (16)	0.0199 (19)	0.0198 (18)	0.0023 (15)	0.0028 (14)	−0.0001 (15)
C14	0.0162 (17)	0.0159 (18)	0.0213 (18)	−0.0012 (15)	0.0042 (15)	−0.0019 (15)
C16	0.0135 (17)	0.0138 (18)	0.0234 (19)	0.0011 (14)	0.0019 (15)	−0.0024 (16)
C9	0.0132 (16)	0.0148 (18)	0.0202 (17)	−0.0013 (14)	0.0021 (13)	0.0006 (14)
C7	0.0127 (17)	0.0193 (18)	0.0222 (19)	0.0027 (15)	0.0018 (15)	−0.0009 (15)
C1	0.023 (2)	0.0185 (19)	0.0181 (18)	0.0058 (16)	−0.0019 (15)	−0.0024 (15)
C4	0.0105 (16)	0.0177 (18)	0.0193 (18)	−0.0005 (14)	0.0010 (13)	−0.0002 (14)
C12	0.0141 (17)	0.0149 (17)	0.0186 (17)	−0.0022 (14)	0.0029 (14)	−0.0008 (14)
C10	0.0142 (17)	0.0151 (17)	0.0214 (19)	−0.0015 (15)	0.0026 (14)	−0.0018 (15)
C5	0.0205 (19)	0.0163 (19)	0.0179 (17)	0.0006 (15)	0.0033 (15)	−0.0023 (15)
C15	0.0183 (18)	0.0163 (18)	0.0179 (18)	−0.0032 (15)	0.0034 (14)	0.0009 (15)
C19	0.033 (3)	0.041 (3)	0.022 (2)	0.009 (2)	0.0062 (19)	−0.005 (2)

### 2-{4-[(E)-(4-Acetylphenyl)diazenyl]phenyl}-1-(5-bromothiophen-2-yl)ethanone Geometric parameters (Å, º)

**Table:**

Br1—C1	1.872 (4)	C8—C7	1.386 (6)
S1—C1	1.713 (5)	C8—C9	1.391 (6)
S1—C4	1.725 (4)	C8—H8	0.9500
O2—C5	1.364 (5)	C17—C16	1.383 (6)
O2—C6	1.402 (5)	C17—C12	1.392 (6)
O1—C5	1.200 (6)	C17—H17	0.9500
O3—O3	0.000 (15)	C11—C10	1.384 (6)
O3—C18	1.214 (6)	C11—H11	0.9500
C2—C1	1.362 (7)	C13—C14	1.385 (6)
C2—C3	1.427 (6)	C13—C12	1.396 (6)
C2—H2	0.9500	C13—H13	0.9500
N1—N1	0.000 (13)	C14—C15	1.403 (6)
N1—N2	1.250 (5)	C14—H14	0.9500
N1—N2	1.250 (5)	C16—C15	1.394 (6)
N1—C9	1.432 (6)	C16—H16	0.9500
N2—N2	0.000 (13)	C9—C10	1.398 (6)
N2—C12	1.432 (5)	C7—H7	0.9500
C3—C4	1.374 (6)	C4—C5	1.467 (6)
C3—H3	0.9500	C10—H10	0.9500
C6—C7	1.379 (6)	C19—H19A	0.9800
C6—C11	1.395 (6)	C19—H19B	0.9800
C18—C15	1.497 (6)	C19—H19C	0.9800
C18—C19	1.510 (6)		
			
C1—S1—C4	90.5 (2)	C17—C16—H16	119.4
C5—O2—C6	116.9 (3)	C15—C16—H16	119.4
C1—C2—C3	111.5 (4)	C8—C9—C10	120.6 (4)
C1—C2—H2	124.3	C8—C9—N1	116.2 (4)
C3—C2—H2	124.3	C10—C9—N1	123.2 (4)
N2—N1—C9	113.6 (4)	C8—C9—N1	116.2 (4)
N2—N1—C9	113.6 (4)	C10—C9—N1	123.2 (4)
N1—N2—C12	113.9 (3)	C6—C7—C8	119.4 (4)
N1—N2—C12	113.9 (3)	C6—C7—H7	120.3
C4—C3—C2	112.1 (4)	C8—C7—H7	120.3
C4—C3—H3	123.9	C2—C1—S1	113.6 (3)
C2—C3—H3	123.9	C2—C1—Br1	127.5 (3)
C7—C6—C11	122.1 (4)	S1—C1—Br1	118.9 (3)
C7—C6—O2	117.0 (4)	C3—C4—C5	130.8 (4)
C11—C6—O2	120.7 (4)	C3—C4—S1	112.3 (3)
O3—C18—C15	120.2 (4)	C5—C4—S1	116.8 (3)
O3—C18—C15	120.2 (4)	C17—C12—C13	120.7 (4)
O3—C18—C19	121.5 (5)	C17—C12—N2	115.4 (4)
O3—C18—C19	121.5 (5)	C13—C12—N2	123.8 (4)
C15—C18—C19	118.3 (4)	C17—C12—N2	115.4 (4)
C7—C8—C9	119.5 (4)	C13—C12—N2	123.8 (4)
C7—C8—H8	120.3	C11—C10—C9	120.1 (4)
C9—C8—H8	120.3	C11—C10—H10	120.0
C16—C17—C12	119.2 (4)	C9—C10—H10	120.0
C16—C17—H17	120.4	O1—C5—O2	125.2 (4)
C12—C17—H17	120.4	O1—C5—C4	124.5 (4)
C10—C11—C6	118.3 (4)	O2—C5—C4	110.2 (4)
C10—C11—H11	120.8	C16—C15—C14	119.0 (4)
C6—C11—H11	120.8	C16—C15—C18	118.9 (4)
C14—C13—C12	119.6 (4)	C14—C15—C18	122.2 (4)
C14—C13—H13	120.2	C18—C19—H19A	109.5
C12—C13—H13	120.2	C18—C19—H19B	109.5
C13—C14—C15	120.4 (4)	H19A—C19—H19B	109.5
C13—C14—H14	119.8	C18—C19—H19C	109.5
C15—C14—H14	119.8	H19A—C19—H19C	109.5
C17—C16—C15	121.2 (4)	H19B—C19—H19C	109.5
			
C9—N1—N2—C12	179.0 (4)	C16—C17—C12—N2	−179.3 (4)
C1—C2—C3—C4	1.3 (6)	C14—C13—C12—C17	1.7 (7)
C5—O2—C6—C7	−126.7 (4)	C14—C13—C12—N2	179.2 (4)
C5—O2—C6—C11	58.0 (6)	C14—C13—C12—N2	179.2 (4)
C7—C6—C11—C10	1.6 (7)	N1—N2—C12—C17	−172.4 (4)
O2—C6—C11—C10	176.7 (4)	N1—N2—C12—C17	−172.4 (4)
C12—C13—C14—C15	−0.3 (7)	N1—N2—C12—C13	10.0 (6)
C12—C17—C16—C15	0.1 (7)	N1—N2—C12—C13	10.0 (6)
C7—C8—C9—C10	2.3 (7)	C6—C11—C10—C9	−0.3 (7)
C7—C8—C9—N1	−178.7 (4)	C8—C9—C10—C11	−1.7 (7)
C7—C8—C9—N1	−178.7 (4)	N1—C9—C10—C11	179.5 (4)
N2—N1—C9—C8	171.0 (4)	N1—C9—C10—C11	179.5 (4)
N2—N1—C9—C8	171.0 (4)	C6—O2—C5—O1	4.3 (7)
N2—N1—C9—C10	−10.1 (6)	C6—O2—C5—C4	−177.0 (4)
N2—N1—C9—C10	−10.1 (6)	C3—C4—C5—O1	174.6 (5)
C11—C6—C7—C8	−1.0 (7)	S1—C4—C5—O1	−3.6 (6)
O2—C6—C7—C8	−176.2 (4)	C3—C4—C5—O2	−4.2 (7)
C9—C8—C7—C6	−1.0 (7)	S1—C4—C5—O2	177.7 (3)
C3—C2—C1—S1	−0.5 (5)	C17—C16—C15—C14	1.3 (7)
C3—C2—C1—Br1	−179.5 (3)	C17—C16—C15—C18	179.8 (4)
C4—S1—C1—C2	−0.3 (4)	C13—C14—C15—C16	−1.2 (6)
C4—S1—C1—Br1	178.8 (3)	C13—C14—C15—C18	−179.6 (4)
C2—C3—C4—C5	−179.7 (5)	O3—C18—C15—C16	−4.3 (7)
C2—C3—C4—S1	−1.5 (5)	O3—C18—C15—C16	−4.3 (7)
C1—S1—C4—C3	1.0 (4)	C19—C18—C15—C16	175.2 (4)
C1—S1—C4—C5	179.5 (4)	O3—C18—C15—C14	174.1 (5)
C16—C17—C12—C13	−1.6 (7)	O3—C18—C15—C14	174.1 (5)
C16—C17—C12—N2	−179.3 (4)	C19—C18—C15—C14	−6.4 (7)

### 2-{4-[(E)-(4-Acetylphenyl)diazenyl]phenyl}-1-(5-bromothiophen-2-yl)ethanone Hydrogen-bond geometry (Å, º)

**Table:**

*D*—H···*A*	*D*—H	H···*A*	*D*···*A*	*D*—H···*A*
C13—H13···N1	0.95	2.47	2.722 (6)	95
C16—H16···O3	0.95	2.49	2.788 (6)	98
C10—H10···N2	0.95	2.45	2.705 (6)	95
